# Gut Microbiota and Exercise-Induced Fatigue: A Narrative Review of Mechanisms, Nutritional Interventions, and Future Directions

**DOI:** 10.3390/nu18030502

**Published:** 2026-02-02

**Authors:** Zhengxin Zhao, Shengwei Zhao, Wenli Li, Zheng Lai, Yang Zhou, Feng Guan, Xu Liang, Jiawei Zhang, Linding Wang

**Affiliations:** 1 Department of Sports, Nanjing University of Finance and Economics HongShan College, Nanjing 211300, China; 9120161014@nufe.edu.cn (Z.Z.); 8220210113@nufe.edu.cn (Y.Z.); 2 Laboratory of Gastrointestinal Microbiology, College of Animal Science and Technology, Nanjing Agricultural University, Nanjing 210095, China; 15850792155@163.com (S.Z.); zhenglai@stu.njau.edu.cn (Z.L.); guanfeng@cau.edu.cn (F.G.); l13951570679@163.com (X.L.); zhang18043055977@163.com (J.Z.); 3 Anhui Provincial Key Laboratory of Zoonoses, Department of Microbiology and Parasitology, School of Basic Medical Sciences, Anhui Medical University, Hefei 230032, China; 2022510051@ahmu.edu.cn

**Keywords:** gut microbiota, exercise-induced fatigue, probiotics, prebiotics, gut–muscle axis, gut–brain axis

## Abstract

**Background:** Exercise-induced fatigue (EIF) impairs performance and recovery and may contribute to overreaching/overtraining and adverse health outcomes. Beyond classical explanations (substrate depletion, metabolite accumulation, oxidative stress), accumulating evidence indicates that the gut microbiota modulates fatigue-related physiology through metabolic, immune, barrier, and neurobehavioral pathways. **Methods:** We conducted a structured narrative review of PubMed and Web of Science covering 1 January 2015 to 30 November 2025 using predefined keywords related to EIF, gut microbiota, recovery, and nutritional interventions. Human studies, animal experiments, and mechanistic preclinical work (in vivo/in vitro) were included when they linked exercise load, microbial features (taxa/functions/metabolites), and fatigue-relevant outcomes. **Results:** Across models, high-intensity or prolonged exercise is consistently associated with disrupted gut homeostasis, including altered community structure, reduced abundance of beneficial taxa, increased intestinal permeability, and shifts in microbial metabolites (e.g., short-chain fatty acids). Evidence converges on four interconnected microbiota-mediated pathways relevant to EIF: (1) energy availability and metabolic by-product clearance; (2) redox balance and inflammation; (3) intestinal barrier integrity and endotoxemia risk; and (4) central fatigue and exercise motivation via microbiota–gut–brain signaling. Nutritional strategies—particularly targeted probiotics, prebiotics/plant polysaccharides, and selected bioactive compounds—show potential to improve fatigue biomarkers and endurance-related outcomes, although effects appear context-dependent (exercise modality, baseline fitness, diet, and baseline microbiota). **Conclusions:** Current evidence supports a mechanistic role of the gut microbiota in EIF and highlights microbiota-targeted nutrition as a promising adjunct for recovery optimization. Future work should prioritize causal validation (e.g., fecal microbiota transplantation and metabolite supplementation), athlete-focused randomized trials with standardized fatigue endpoints, and precision approaches that stratify individuals by baseline microbiome features and training load.

## 1. Introduction

Exercise-induced (EIF) fatigue, defined as the body’s inability to maintain a specific functional level or adhere to a predetermined intensity, is a common physiological response to athletic training and physical exertion [1]. While mild daily fatigue is reversible through rest, prolonged or excessive training can induce overtraining syndrome or chronic fatigue. In this review, “high-intensity exercise” is used as an umbrella term covering workloads that approach or exceed ventilatory/lactate thresholds (e.g., near-maximal intervals), prolonged endurance exercise with substantial thermal/physiological strain, or repeated high-load training that induces measurable performance decrement and/or marked biochemical stress responses [2]. These conditions compromise athletic performance and training efficacy while increasing the risk of immune suppression and endocrine disorders, thereby threatening both athlete competitiveness and general public health [3,4]. Conventionally, fatigue is attributed to substrate depletion (e.g., ATP, glycogen), metabolite accumulation (e.g., lactate, blood urea nitrogen), oxidative stress, ion imbalances, and neuro-endocrine-immune (NEI) network dysregulation [5]. However, recent advances in microecology have elucidated the pivotal role of the gut microbiota in regulating fatigue, offering novel insights into its mechanisms and potential interventions.

The gut microbiota is a complex microbial ecosystem that colonizes the human gastrointestinal tract, comprising bacteria, fungi, viruses, and other microorganisms. It plays a pivotal role in essential physiological processes, including host energy metabolism, nutrient absorption, immune regulation, and the maintenance of barrier function [6]. Research has established a close bidirectional regulatory relationship between the gut microbiota and the motor system—termed the “gut–muscle axis”—as well as the central nervous system via the “gut–brain axis,” which collectively influence motor capacity and fatigue perception [7,8]. Numerous studies have demonstrated that high-intensity exercise can induce intestinal dysbiosis, characterized by a reduction in the abundance of beneficial microbes, the proliferation of pathogenic bacteria, increased intestinal permeability, and an elevated risk of endotoxemia [6,9]. Conversely, the gut microbiota significantly impacts exercise capacity, fatigue performance, and immune function. Currently, research regarding the relationship between gut microbiota and EIF has coalesced around several core directions: firstly, the regulation of energy metabolism, specifically how specific microbial taxa optimize host glycogen reserves and accelerate the clearance of metabolic waste (such as lactate) by producing metabolites like short-chain fatty acids (SCFAs) [10,11,12]; secondly, the attenuation of oxidative stress and inflammatory responses, where the microbiota and its metabolites enhance the host’s antioxidant capacity and inhibit exercise-induced systemic low-grade inflammation; thirdly, the maintenance of intestinal barrier function by consolidating the physical mucosal barrier, thereby preventing the translocation of harmful substances (e.g., endotoxins) into the bloodstream and mitigating negative cascade reactions along the “bacteria-gut–muscle” axis [13]; and finally, the regulation of central fatigue via the “gut–brain” axis, through which the microbiota influences the synthesis and metabolism of neurotransmitters, thereby modulating emotion, motivation, and the perception of central fatigue [7]. Together, these research avenues delineate a complex regulatory network, highlighting the immense potential of the gut microbiota as a novel target for anti-fatigue interventions.

Based on these regulatory relationships, targeted modulation of the gut microbiota using nutritional and biological strategies has emerged as a focal point in exercise fatigue research. At present, strategies aimed at alleviating EIF via the gut microbiota primarily encompass probiotic interventions, supplement prebiotics (plant polysaccharides and extracts), protein peptides, and other nutrients, as well as adjust dietary patterns. Probiotics refer to live microorganisms that, when administered in adequate amounts, confer a health benefit on the host, whereas prebiotics are selectively utilized substrates (often indigestible fibers/polysaccharides) that promote beneficial microbial functions [14]. These represent distinct strategies with different mechanistic targets and time courses of action. Concurrently, mechanistic research has transitioned from merely describing microbiota structure to delving into deep metabolic pathway regulation. Drawing upon cutting-edge research from recent years, this article systematically reviews progress in this field, focusing on the correlations, mechanistic pathways, and intervention strategies linking gut microbiota to EIF, with the aim of providing a reference for future research and application.

## 2. Methods

### Literature Search Strategy

A comprehensive literature search was conducted for this narrative review, covering publications from 1 January 2015 to 30 November 2025. Databases including PubMed and Web of Science were queried using specific keyword combinations. The primary search strings included:

- PubMed: (fatigue OR “exercise-induced fatigue” OR exhaustion OR overtraining OR recovery) AND (“gut microbiota” OR microbiome OR “intestinal flora” OR dysbiosis) AND (probiotic* OR prebiotic* OR polysaccharide* OR nutrition* OR supplement* OR “dietary intervention”);
- Web of Science: TS = (fatigue OR “exercise-induced fatigue” OR exhaustion OR overtraining OR recovery) AND TS = (“gut microbiota” OR microbiome OR “intestinal flora” OR dysbiosis) AND TS = (probiotic* OR prebiotic* OR polysaccharide* OR nutrition* OR supplement* OR “dietary intervention”).

**Figure 1:**
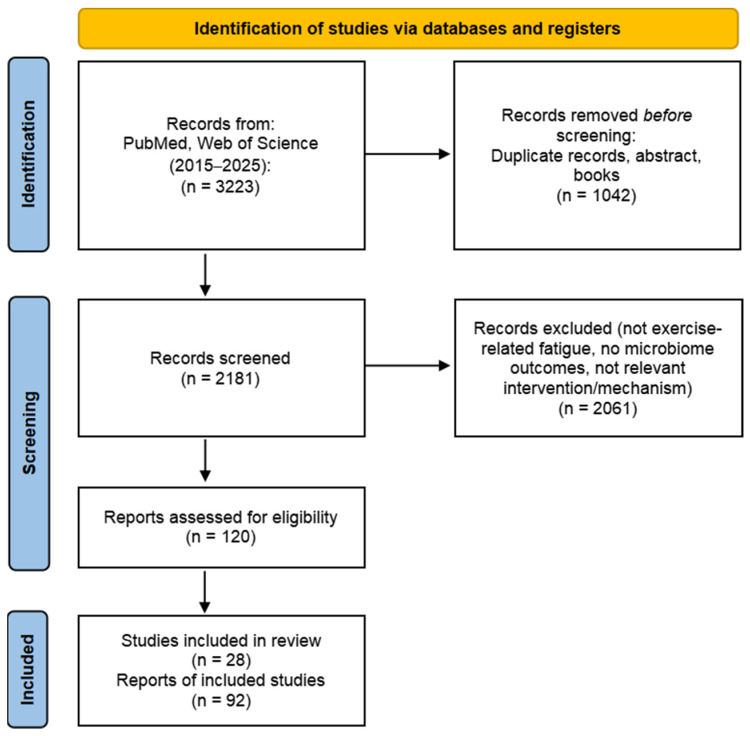
PRISMA diagram illustrating the literature search and selection process.

Our search resulted in 3223 total references, and 120 papers were within our scope of interest. The selection process is illustrated using the PRISMA 2020 flow diagram (Figure 1) [15]. Inclusion criteria encompassed randomized controlled trials, observational studies, case reports, and preclinical models (both in vivo and in vitro). This article is a structured narrative review rather than a PRISMA-guided systematic review; therefore, protocol preregistration (e.g., PROSPERO) and formal risk-of-bias grading were not undertaken. A formal meta-analysis was not performed due to substantial heterogeneity in exercise protocols, populations/species, microbiome assessment methods, intervention types/doses/durations, and outcome definitions of fatigue (performance tests vs. biochemical markers vs. perceived fatigue), which precluded meaningful quantitative pooling.

## 3. Characteristics of Changes in Gut Microbiota Under Exercise-Induced Fatigue

### 3.1. Changes in Gut Microbiota Diversity

Exercise-induced fatigue, particularly resulting from high-intensity or excessive training, significantly alters gut microbial composition, primarily manifesting as shifts in diversity and community structure [16,17]. Microbial diversity, a key indicator of gut health, is categorized into α-diversity (within-community richness and evenness) and β-diversity (between-sample structural differences) [18]. Wu observed significantly higher α-diversity (Shannon index) in fatigued rats using a slope treadmill model, indicating increased species richness [19]. MeiHua [9] reported no significant alterations in α or β-diversity in young boxers post-fatigue, although a downward trend was noted. Bennett [20] identified a non-linear, complex association between α-diversity and markers of gastrointestinal injury in endurance athletes under heat stress. Furthermore, baseline fitness levels (trained vs. sedentary) likely influence outcomes; athletes adapted to high-load training may possess an “adaptive homeostasis,” resulting in fatigue response patterns distinct from those of experimental animals. Despite inconsistencies regarding α-diversity trends, a consensus exists: high-intensity EIF functions as a potent environmental stressor disrupting gut microbial equilibrium. Significant shifts in microbial community structure (β-diversity) have been consistently observed across diverse contexts, including acute exhaustive exercise, long-term endurance training, and environmental heat stress [9,20,21].

### 3.2. Changes in Gut Microbiota Structure

In addition to changes in overall abundance, EIF drives specific alterations in bacterial abundance linked to the pathophysiology of fatigue. *Firmicutes* and *Bacteroidetes* constitute the dominant phyla within the gut ecosystem. Evidence suggests that microbial composition and functional metabolites significantly influence host health status, fatigue, and recovery. Consequently, the *Firmicutes*/*Bacteroidetes* (F/B) ratio is frequently utilized as a health metric, though its interpretation requires consideration of inter-individual variability [22,23]. Numerous studies have documented significant F/B ratio fluctuations in animal models of EIF. For instance, Huang [24] observed that stigmasterol treatment alleviated fatigue in rats, concurring with a decreased F/B ratio (reduced *Firmicutes*, increased *Bacteroidetes*), suggesting a potential benefit of this profile. Conversely, contradictory trends have been reported. In mouse models exhibiting severe fatigue or overtraining, intense physiological stress often precipitates a decline in *Bacteroidetes* and a compensatory rise in *Firmicutes* [25]. These shifts frequently correlate with compromised barrier function and systemic inflammation.

At the genus level, distinct patterns emerge among key bacterial taxa. In animal model experiments, Populations of traditional probiotics, such as *Lactobacillus* and *Bifidobacterium*, typically decline under fatigue conditions [26,27]. This reduction may compromise intestinal barrier integrity and diminish the production of beneficial metabolites like short-chain fatty acids (SCFAs), potentially exacerbating fatigue. Conversely, the abundance of potentially pathogenic or opportunistic taxa may increase. For example, Yuan [28] observed an expansion of the inflammation-associated phylum *Proteobacteria* in an overtrained mouse model. Furthermore, specific genera exhibit positive correlations with athletic performance and recovery capacity. Notably, elite athletes possess enriched populations of *Veillonella*. This genus metabolizes exercise-induced lactate into propionate, providing an additional energy substrate for the host. This exemplifies “gut–muscle axis” interaction, highlighting the potential of specific microbial communities to enhance performance and accelerate recovery [29]. Thus, fatigue-induced microbiota shifts are not random stochastic events but reflect targeted fluctuations in functional communities. Collectively, these alterations constitute the microbiological underpinnings of EIF.

## 4. The Primary Mechanism of Intestinal Flora Regulating Exercise-Induced Fatigues

### 4.1. Regulate Energy Metabolism

**Figure 2:**
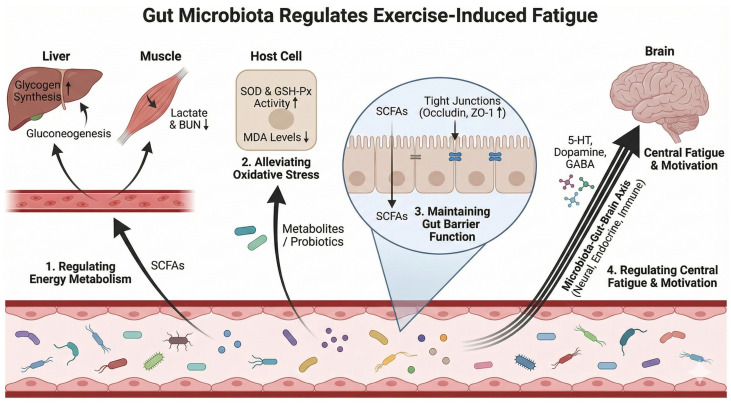
The main pathways through which gut microbiota affects exercise-induced fatigue. BUN, Blood Urea Nitrogen; SCFAs, short-chain fatty acids; SOD, Superoxide Dismutase; GSH-Px, Glutathione peroxidase; MDA, malondialdehyde.

Substrate depletion and metabolic waste accumulation are fundamental etiologies of EIF [30,31]. Accumulating evidence suggests that modulating the gut microbiota effectively optimizes host energy metabolism and delays fatigue onset. Mechanistically, specific microbial taxa and their metabolites enhance host energy reserves (Figure 2). For instance, supplementation with *Lactiplantibacillus plantarum* or ginseng extract has been shown to significantly elevate hepatic and muscle glycogen levels in mice following exhaustive exercise [32,33]. This effect is likely mediated by microbial-derived short-chain fatty acids (SCFAs). Beyond serving as the primary energy source for colonocytes, SCFAs enter systemic circulation to facilitate hepatic and muscular gluconeogenesis and glycogenesis [34]. Specifically, ginseng extract exerts anti-fatigue effects by enriching SCFA-producing bacteria, thereby upregulating glycogen synthesis [33]. Similarly, distinct probiotic strains, including *Lactobacillus rhamnosus* SDSP202418 and *Pediococcus pentosaceus* YF01, bolster glycogen reserves and prolong the endurance performance of mice [35,36].

Concurrently, the gut microbiota facilitates the clearance of fatigue-associated metabolites, including lactate and blood urea nitrogen (BUN). High-intensity exercise induces the rapid accumulation of lactate and ammonia in blood and muscle tissue, precipitating acidosis and fatigue. Supplementation with *Weizmannia coagulans* BC99, *L. rhamnosus* SDSP202418, or fermented deer blood significantly attenuates serum lactate and BUN levels in mice post-exercise [35,37,38]. Mechanistically, this involves the modulation of host metabolic pathways. For instance, resveratrol improves fatigue biomarkers (e.g., glucose, lactate, BUN, LDH, CK) and antioxidant enzyme activity (CAT, GSH-Px) by enhancing microbial diversity and modulating taxa involved in inflammation and fatty acid metabolism [25]. Moreover, certain probiotic interventions modulate lactate dehydrogenase (LDH) activity, underscoring the microbiota’s direct influence on metabolic waste clearance [32,36].

### 4.2. Reduce Oxidative Stress and Inflammatory Response

Intense exercise disrupts redox homeostasis, causing excessive reactive oxygen species (ROS) production. This triggers oxidative stress, cellular damage, and inflammation—key drivers of EIF and muscle injury [39,40]. The gut microbiota modulates host redox homeostasis and inflammatory responses via multiple mechanisms. Mechanistically, probiotics and their metabolites exhibit direct antioxidant properties. For instance, supplementation with *Pediococcus pentosaceus* YF01 significantly enhances antioxidant enzyme activity (e.g., SOD, GSH-Px) in mice while reducing lipid peroxidation markers like malondialdehyde (MDA) [35]. In a murine model of fatigue, Periplaneta americana glycoprotein (PAG) mitigates fatigue by restoring microbial homeostasis and increasing SCFA production, thereby upregulating antioxidant expression (SOD, GSH-Px) and protecting cells against exercise-induced oxidative damage [41].

High-intensity exercise increases intestinal permeability, facilitating the translocation of microbial endotoxins (e.g., lipopolysaccharide/LPS) into the bloodstream [6]. This process activates the TLR4 signaling pathway, triggering systemic low-grade inflammation. Using 16S rRNA sequencing in endurance athletes under heat stress, Bennett et al. [20] identified correlations between specific taxa, gastrointestinal injury, thermoregulation, and inflammatory responses. Supplementation with specific probiotics or dietary components can effectively mitigate these effects. Wang et al. confirmed through animal models that specific pathogenic taxa correlate with inflammation and metabolic dysregulation, whereas beneficial clusters are associated with enhanced metabolism [42]. Similarly, ginseng extract downregulates serum inflammatory markers (IL-6, IL-1β, CRP) in exercised rats, an effect linked to microbiota modulation and SCFA production [33].

### 4.3. Maintain Intestinal Barrier Function

Beyond energy metabolism, oxidative stress, and immune regulation, the gut microbiota modulates systemic inflammation and neuro-endocrine pathways by maintaining intestinal barrier integrity. These interactions, mediated by the gut–muscle and gut–brain axes, significantly influence EIF and recovery. Evidence indicates that exercise alters microbial composition and metabolite profiles, subsequently impacting barrier function, systemic inflammation, and energy metabolism. Conversely, compromised barrier integrity facilitates the translocation of endogenous endotoxins (e.g., LPS) into circulation, driving inflammatory cascades and metabolic imbalances that manifest as heightened fatigue and delayed recovery [6,43,44]. Experiments in animal models have demonstrated that the co-administration of *Clostridium butyricum* and chitosan oligosaccharides stimulates the production of short-chain fatty acids and upregulates the expression of tight junction proteins (e.g., Occludin and ZO-1), thereby strengthening intestinal barrier function [45]. While exhaustive exercise compromises the intestinal barrier in mice, supplementation with neomannuronan-agglutaronan-tetraose (NAT) restores intestinal morphology and function via microbiota modulation, thereby exerting anti-fatigue effects [46]. In a murine model of fatigue, *Brassica rapa* L. supplementation alleviates inflammation by inhibiting LPS-induced NF-κB pathway activation and curbing Gram-negative bacterial proliferation, thereby mitigating fatigue [42]. Emerging evidence suggests that plant polysaccharides and natural products contribute to barrier maintenance and fatigue alleviation by modulating microbial communities and their metabolites [8,47]. Consequently, microbial metabolites are considered core mediators linking barrier homeostasis to fatigue regulation.

### 4.4. Regulation of Central Fatigue and Exercise Motivation

EIF encompasses both peripheral muscular fatigue and central nervous system (CNS) fatigue, the latter manifesting as cognitive deficits, mood disturbances, and diminished exercise motivation [48,49,50]. The gut microbiota profoundly influences central fatigue via the “microbiota–gut–brain axis,” a bidirectional communication network linking the enteric and central nervous systems. This crosstalk involves complex neural, endocrine, and immunological pathways (Table 1). Microbial taxa synthesize or modulate neurotransmitters—including serotonin (5-HT), dopamine, and γ-aminobutyric acid (GABA)—that regulate cerebral function and emotional states [51]. For instance, a murine study identified a specific gut–brain pathway that enhances performance by potentiating dopamine signaling during physical exertion. Specifically, microbiome-dependent production of endocannabinoid metabolites activates TRPV1 sensory neurons, subsequently elevating dopamine levels in the nucleus accumbens. Activation of this pathway enhances running performance, whereas microbiome depletion, peripheral endocannabinoid receptor inhibition, spinal afferent ablation, or dopamine blockade impairs capacity [7].

## 5. Therapeutic Strategies for Modulating the Gut Microbiota to Mitigate Exercise-Induced Fatigue

### 5.1. Probiotic Interventions

Probiotic intervention represents the most direct and extensively researched strategy for modulating the gut microbiota to alleviate EIF (Table 2). Accumulating evidence demonstrates that specific strains enhance energy metabolism and attenuate exercise-induced inflammation by restoring microbial homeostasis [52,53]. Single-strain interventions, particularly involving *Lactobacillus* and *Bifidobacterium*, have exhibited significant anti-fatigue potential. For instance, both live and heat-inactivated *Lactiplantibacillus plantarum* TWK10 improve athletic performance and body composition in humans [54]. Similarly, in murine models, *L. plantarum* CCFM1280 significantly extends exhaustive swimming time, attenuates lactate accumulation, and elevates glycogen reserves [32]. In a murine model of fatigue, *Lacticaseibacillus paracasei* NB23 (1 and 3 × 10^10^ CFUs/human/day) optimizes host energy metabolism and accelerates metabolite clearance [55]. Beyond common lactobacilli, novel probiotics such as *Limosilactobacillus reuteri* ID-D01 have demonstrated robust anti-fatigue properties, increasing maximum running distance in rats [10]. Moreover, *Lactobacillus rhamnosus* SDSP202418 prolongs the endurance performance of mice by upregulating slow-twitch muscle fiber mRNA expression and downregulating inflammatory markers [35]. Beyond single-strain applications, multi-strain formulations have gained attention for their potential synergistic effects. A double-blind study on humans revealed that a combination of *Lactococcus lactis* subsp. LY-66 and *L. plantarum* PL-02, coupled with regular training, significantly enhances maximal oxygen uptake (VO_2_max) and performance [56]. Similarly, a synbiotic preparation containing *Pediococcus acidilactici* and *L. plantarum* was shown to improve the intestinal environment in endurance runners, primarily targeting gastrointestinal distress [57].

### 5.2. Plant Polysaccharides and Extracts

Distinct from probiotic interventions, the use of plant polysaccharides and extracts as prebiotics to modulate endogenous microbiota represents a pivotal strategy for fatigue management (Table 3). These indigestible substrates undergo microbial fermentation, yielding beneficial metabolites such as short-chain fatty acids (SCFAs) [60]. Notably, polysaccharides derived from Traditional Chinese Medicine (TCM) have garnered significant attention for their anti-fatigue properties. Polysaccharides from Millettia speciosa Champ. (MCP) have been shown to alleviate fatigue induced by excessive exercise. In animal models, MCP modulates microbial composition—enriching beneficial taxa—thereby enhancing antioxidant activity and improving biochemical markers [61,62]. In another animal experiment, *Polygonati rhizoma*, a medicinal and edible plant, exhibits anti-fatigue effects attributed to its polysaccharide component (PRP), an effect linked to microbiota modulation [63]. Ginseng extends the weight-bearing swimming time of mice and promotes the abundance of SCFA-producing bacteria. This elevates serum SCFA levels, facilitates glycogen synthesis, and inhibits inflammation [33]. A recent animal model studies distinguish the synergistic anti-fatigue roles of ginseng polysaccharides and saponins, which operate via distinct mechanisms [12]. In a murine model of fatigue, fermentation enhances the bioavailability of ginsenosides, thereby augmenting their anti-fatigue efficacy [27,64]. Beyond TCM, polysaccharides and extracts from diverse plant sources demonstrate significant anti-fatigue potential. In exhaustive swimming experiments with mice, Zingiber officinale polysaccharide (ZOPA) exerts anti-fatigue effects via the “gut–muscle” axis [13]. Functioning as a prebiotic, *Tuber indicum* polysaccharide reduces blood lactate and modulates the microbiota in fatigued mice [65]. In overtraining animal models, konjac glucomannan (KGM) enhances gut microbial health, endurance, and strength [66]. Collectively, these findings underscore the potential of plant-derived prebiotics in alleviating fatigue, particularly through the regulation of microbial metabolites.

### 5.3. Proteins, Peptides, and Other Nutrients

In addition to traditional probiotics and prebiotics, the roles of proteins, peptides, and other novel nutritional supplements in modulating intestinal flora and alleviating exercise fatigue have increasingly garnered scholarly attention. These substances primarily exert their effects by providing metabolic substrates or directly regulating the intestinal microenvironment. For instance, proteins and their hydrolysates are critical for muscle repair and energy provision. An animal study indicates that whey protein hydrolysate (WPH) is superior to whey protein concentrate (WPC) in delaying fatigue. This advantage is likely attributed to its high content of low molecular weight peptides and branched-chain amino acids, an effect potentially linked to the modulation of intestinal flora [70]. Notably, combining probiotics (such as *Weizmannia coagulans* BC99, 1 × 10^7^ CFU/g·bw/day) with protein (2.5 mg/g·bw/day) supplements can significantly enhance protein digestive enzyme activity and improve exercise endurance of mice. This “probiotics + protein” synergistic strategy offers new insights for sports nutrition research [38]. Furthermore, in animal experiments utilizing a compound derived from marine by-products—specifically grouper bone hydrolysate and Undaria pinnatifida extract (referred to as GU)—gut microbiota profiling revealed distinct shifts in community composition. Notably, the GU-treated group (6150 mg/kg/day) exhibited an increased abundance of beneficial taxa such as *Lactobacillus* and *Muribaculum*. These alterations reflect the prebiotic activity of seaweed-derived polysaccharides, which promote a healthier gut microbial profile [71].

Interventions involving other specific bioactive nutrients have also demonstrated unique effects. For instance, soybean-derived stigmasterol exhibited significant anti-fatigue efficacy in rats with exercise-induced fatigue (EIF), as evidenced by prolonged time to exhaustion and reduced levels of myocardial injury markers. Mechanistically, stigmasterol was found to upregulate the expression of key energy metabolism-related genes (e.g., SIRT1, PGC-1α, HK2, MCT-1, and NRF-1). Furthermore, it increased glycogen and glucose (Glu) reserves while decreasing lactic acid (LA) and blood urea nitrogen (BUN) accumulation, thereby optimizing energy metabolic status and alleviating EIF [24]. Ethanol-extracted propolis (EEP) alters the composition and structure of intestinal microorganisms by increasing the abundance of butyrate-producing bacteria and reducing harmful bacteria, thereby significantly improving exercise endurance and anti-fatigue effects in mice [72]. As a cereal rich in β-glucan, highland barley intake in varying proportions significantly affects anti-fatigue capacity, energy metabolism, and intestinal flora composition in mice [73]. Studies have found that camellia oil (CO) significantly increased the relative abundances of Alistipes, *Alloprevotella*, *Lactobacillus*, and Muribaculaceae, while decreasing that of *Dubosiella*, thereby ameliorating gut microbiota dysbiosis in mice with EIF. Furthermore, the specific microbial taxa modulated by CO were significantly correlated with several fatigue-related parameters, indicating that CO may alleviate EIF in mice by regulating intestinal microbial composition [74]. Collectively, these studies suggest that anti-fatigue nutritional interventions are shifting from single-nutrient supplementation toward the use of diverse bioactive substances to holistically regulate intestinal microecology.

### 5.4. Other Intervention Schemes

In addition to traditional probiotics and prebiotics, emerging intervention strategies and integrative perspectives are continuously evolving, providing broader avenues for regulating intestinal flora and combating fatigue. These strategies encompass specific dietary patterns, non-nutritive substance supplementation, and targeted medical treatments, collectively pointing toward a more personalized and multidimensional approach to intervention. For instance, the combined intervention of time-restricted fasting (TRF) and nicotinamide mononucleotide (NMN) has been demonstrated to enhance the athletic performance of mice by activating mitochondrial function and modulating intestinal flora [75]. This suggests that integrating dietary rhythms with specific nutrient supplementation may yield synergistic effects. Similarly, hydrogen water, acting as an antioxidant, has been found to improve endurance exercise performance of rats by regulating intestinal flora and activating the Pparγ/PGC-1α pathway [76], highlighting the potential of non-traditional dietary components. Regarding medical treatments, Wu [19] demonstrated through animal model research that low-intensity pulsed ultrasound therapy can ameliorate intestinal permeability, inflammatory responses, and microbial diversity in rats with EIF. By regulating the IL-13 and NF-κB signaling pathways, this therapy promotes energy metabolism and enhances exercise performance, thereby providing a theoretical basis for the microbial regulation of exercise endurance.

## 6. Conclusions and Discussion

This review systematically summarizes the interplay between the gut microbiota and exercise-induced fatigue (EIF) based on current evidence. Key findings from the results section are as follows: (1) High-intensity exercise-induced fatigue disrupts gut microbial homeostasis, which is characterized by inconsistent α-diversity alterations (species- and model-dependent) but consistent β-diversity shifts across human and animal studies; (2) EIF drives distinct taxonomic changes in the gut microbiota, including reduced abundances of beneficial taxa (*Lactobacillus*, *Bifidobacterium*), expanded populations of pathogenic/opportunistic taxa (*Proteobacteria*), and context-dependent fluctuations in the *Firmicutes*/*Bacteroidetes* (F/B) ratio; (3) The gut microbiota modulates EIF through four interconnected regulatory pathways: optimization of energy metabolism, attenuation of oxidative stress and inflammation, maintenance of intestinal barrier integrity, and modulation of central fatigue via the microbiota–gut-brain axis; (4) Probiotics (single-/multi-strain), plant polysaccharides, and protein peptides effectively mitigate EIF by targeting gut microbial composition and metabolic profiles, with their efficacy being dependent on intervention dosage and duration.

The inconsistent α-diversity changes observed in EIF models—such as increased α-diversity in rats [19] versus no significant changes in boxers [6,9]—can be attributed to two key factors: (1) Differences in exercise protocols: The slope treadmill model [19] involved 12 weeks of progressive chronic training, while the boxer study [6,9] adopted a single bout of acute exhaustive sparring, suggesting that α-diversity responses to EIF may be time-dependent, varying between chronic and acute fatigue states. (2) Differences in host baseline characteristics: Fundamental disparities exist between experimental subjects (humans versus animal models) per se. In addition, elite athletes (e.g., boxers) may develop a state of adaptive homeostasis in the gut microbiota due to long-term systematic training, whereas naive laboratory animals (rats/mice) exhibit more pronounced α-diversity shifts under exercise stress. These factors underscore the necessity for standardized exercise protocols and stratified analysis by training status in future EIF-microbiota research.

At the phylum level, inconsistent alterations in the F/B ratio across EIF models—decreased in stigmasterol-treated fatigued rats [24] versus increased in overtrained animal models [25]—challenge its validity as a universal biomarker for fatigue. This highlights the need for cautious interpretation of the F/B ratio, which cannot be regarded as a universal indicator of fatigue status. The direction and magnitude of F/B ratio changes are influenced by multiple variables, including dietary composition, host age, training status, exercise modality, and analytical pipelines; moreover, the recent literature has questioned the standalone interpretability of this ratio across different physiological conditions. Therefore, fatigue-related inferences should prioritize pathway-level functional outputs (e.g., short-chain fatty acid production, intestinal barrier markers, inflammatory signatures) rather than sole reliance on the F/B ratio.

The microbiota–gut–brain axis represents a novel research frontier in the regulation of central fatigue. Specific microbial taxa synthesize or modulate key neurotransmitters (serotonin, dopamine, γ-aminobutyric acid) that regulate cerebral function and exercise motivation [51]. A pivotal example is the microbiome-dependent production of endocannabinoid metabolites, which activate TRPV1 sensory neurons and elevate dopamine levels in the nucleus accumbens—thereby enhancing running performance and exercise motivation [7,8]. This pathway demonstrates that the gut microbiota influences not only the physiological capacity for exercise (“can”) but also the psychological motivational drive for physical activity (“wants”). Consequently, targeted modulation of this axis via probiotics or other therapeutic approaches constitutes a novel strategy to alleviate central fatigue and enhance exercise motivation.

Probiotic interventions for EIF have evolved from simple gut microbial regulation to complex strategies targeting systemic metabolic and immunomodulatory processes. For example, *Lactobacillus rhamnosus* SDSP202418 [35,36] upregulates the expression of slow-twitch muscle fiber-related genes, while *Lacticaseibacillus paracasei* NB23 [55] accelerates the clearance of exercise-induced metabolic byproducts. Furthermore, probiotic supplementation modulates post-exercise cytokine profiles, although its clinical efficacy in human populations requires further systematic evaluation [54]. Heat-inactivated probiotics (e.g., *Lactobacillus plantarum* TWK10 [54]) also exhibit anti-fatigue efficacy, suggesting that microbial metabolites or cell wall components may contribute to these beneficial effects, rather than viable bacterial cells alone. Multi-strain probiotic formulations offer synergistic anti-fatigue benefits: the combination of *Lactococcus lactis* subsp. LY-66 and *Lactobacillus plantarum* PL-02 [56] enhances maximal oxygen uptake (VO_2_max) more effectively than single-strain interventions, likely due to complementary microbial targeting and synergistic metabolite production. A key limitation of current probiotic research, however, is strain specificity—interventions effective in animal models (e.g., *Lactobacillus plantarum* CCFM1280 [32,33] in mice) may not directly translate to humans, which highlights the critical need for well-designed human clinical trials to validate their efficacy.

Beyond microbiota-targeted interventions, numerous traditional ergogenic supplements (e.g., carnitine, creatine) improve physical exercise performance. For instance, carnitine supplementation (2 g/dL for 4 weeks at rest) significantly altered resting maximal oxygen consumption (VO_2_) (*p* < 0.005) and serum free and total carnitine levels at rest and post-exercise (*p* < 0.001) [77]. A major challenge in this field, however, is identifying ergogenic supplements that exert direct or indirect modulatory effects on gut microbial composition and function, and establishing causal links between these microbiome alterations and clinically meaningful fatigue endpoints. This emphasizes the need for integrative clinical trials that simultaneously capture intervention parameters, gut microbiome functional readouts, and standardized fatigue phenotypes.

## 7. Future Work

Nonetheless, current research still faces notable limitations. Most studies merely elucidate the “correlation” between microbial alterations and fatigue amelioration, lacking a comprehensive molecular causal pathway linking microbiota and metabolites to the regulation of physiological fatigue processes. Furthermore, insufficient attention has been directed toward inter-individual variability in intervention responses arising from host genetic backgrounds and baseline microbial community structures. The translation of findings from animal models to humans—particularly elite athletes—remains constrained by the paucity of large-scale, high-quality randomized controlled clinical trials. Future research should move from association to causality by combining microbiome depletion or fecal microbiota transplantation with targeted metabolite supplementation and pathway readouts. In humans, adequately powered trials—ideally stratified by baseline microbiome features and training status—should adopt standardized fatigue endpoints that distinguish acute fatigue from chronic/overtraining-related phenotypes, and incorporate objective physiological measures (e.g., CPET-derived indices) alongside symptom and biomarker panels [78,79]. Finally, athlete-focused studies are needed to clarify whether microbiota-targeted strategies translate to elite performance contexts compared with the general population, and to develop robust indicators of central fatigue (e.g., motivation-related measures, neuroendocrine readouts, and validated perceptual/cognitive metrics). Ultimately, the objective is to facilitate the practical application of anti-fatigue strategies targeting the gut microbiota, transitioning from the laboratory to real-world practice. This will provide scientific support for optimizing athletes’ competitive status and preventing chronic fatigue in the general population, while fostering interdisciplinary innovation in sports medicine and microecology.

## Figures and Tables

**Table 1:** Mechanistic pathways linking gut microbiota to exercise-induced fatigue.

Pathway	Microbial Features/Targets	Key Mediators (Examples)	Fatigue-Relevant Outcomes	Evidence Base	Representative Refs
Energy metabolism & metabolite clearance	↑ SCFA-producing bacteria; probiotic strains (e.g., *Lactiplantibacillus plantarum*; *Lactobacillus rhamnosus* SDSP202418; *Pediococcus pentosaceus* YF01; *Weizmannia coagulans* BC99); functional shifts supporting glycogen synthesis and lactate handling	SCFAs (acetate/propionate/butyrate); gluconeogenesis/glycogenesis signaling; LDH modulation	↑ hepatic/muscle glycogen; ↓ serum lactate & BUN; ↓ LDH/CK (when reported); prolonged endurance/exhaustion time	Mainly animal + intervention studies; some mechanistic links	[25,30,31,32,33,34,35,37,38]
Oxidative stress & inflammatory regulation	Probiotics/food bioactives restoring microbial homeostasis; reduced pathogenic taxa clusters; improved SCFA output; taxa–inflammation associations in athletes under heat stress	ROS/redox enzymes (SOD, GSH-Px, CAT); lipid peroxidation (MDA); cytokines (IL-6, IL-1β, CRP); LPS–TLR4 signaling	↓ oxidative damage (↓ MDA); ↑ antioxidant enzymes; ↓ systemic inflammation; improved recovery/fatigue biomarkers	Animal intervention + limited human association evidence	[6,20,33,35,39,40,42]
Intestinal barrier integrity & endotoxemia risk	Barrier-supportive microbiota; ↑ butyrate producers; suppression of Gram-negative overgrowth (context-dependent); polysaccharides/natural products modulating communities and metabolites	Tight junction proteins (Occludin, ZO-1); SCFAs (butyrate); LPS translocation; NF-κB pathway	↓ intestinal permeability; ↓ endotoxemia-driven inflammation; improved fatigue tolerance and recovery	Primarily animal + mechanistic; supportive literature on permeability	[6,8,43,44,45,46,47]
Central fatigue & exercise motivation (gut–brain axis)	Microbiota-dependent neuromodulatory pathways; taxa influencing neurotransmitter metabolism; microbiome-dependent endocannabinoid signaling activating sensory neurons	Neurotransmitters (5-HT, dopamine, GABA); endocannabinoid metabolites; TRPV1 sensory neurons; nucleus accumbens dopamine signaling	↑ exercise motivation/capacity; mitigation of CNS-related fatigue symptoms; improved performance via motivational drive	Mechanistic evidence (animal)	[7,48,49,50,51]

**Table 2:** The alterations in the composition of gut bacteria following the administration of probiotics.

Bacteria Strain	Subject(s)	Dose	Duration	Changed Abundance of Gut Microbiota	Main Conclusion	Ref
*L. reuteri* ID-D01	Sprague-Dawley rats	3.6 × 10^7^, 1.82 × 10^9^ CFU/g	53 days	*Verrucomicrobia*, *Ruminococcaceae*, *Lachnospiraceae*, *Akkermansia* spp. ↑	Enhance endurance, reduce fatigue, modulate gut microbiota, promote SCFA production	[10]
*L. brevis* GKEX	Male ICR mice	4 × 10^11^ CFU/g	28 days	*Anaeroplasma*, *Roseburia*, *Blautia*, *Eubacterium*, *Christensenella minuta* ↑	Improve endurance, regulate lactic acid metabolism, and boost beneficial bacteria	[58]
*L. rhamnosus* SDSP202418	Kunming mice	1 × 10^9^ CFU/mL	3 weeks	*Lactobacillus*, *Alloprevotella*, *Odoribacter*, *Prevotellaceae_UCG-001*, *Alistipes*, and *Anaeroplasma* ↑; *Candidatus_Saccharimonas* ↓	Enhance exercise performance/muscle mass, increase beneficial bacteria, improve body composition/gut health	[35]
*P. pentosaceus* YF01	Male Kunming mice	1 × 10^8^ CFU/mL	4 weeks	*Lactobacillus*, *Lachnospiraceae*, and *Burkholderiales* ↑	Prolong exhaustion time, ameliorate oxidative stress, regulate muscle/liver gene expression, and elevate probiotic abundance	[36]
*W. coagulans* BC99	Male Kunming mice	1 × 10^6^, 1 × 10^7^, 1 × 10^8^ CFU/g	6 weeks	*Roseburia*, *Mucispirillum*, *Rikenella*, *Kineothrix* ↑	+Protein: elevate protease activity, antioxidant capacity, microbiota diversity; enhance endurance, reduce fatigue	[38]
L. plantarum TWK10	Healthy men and women	1 × 10^11^ CFU/capsule	6 weeks	*Akkermansiaceae*, *Prevotellaceae* ↑	Viable/heat-inactivated TWK10: improve exercise capacity, anti-fatigue response; viable form: better muscle gain/fat loss, distinct microbiota/metabolic pathway changes	[54]
*L. salivarius* UCC118	trained endurance athletes	2 × 10^8^ CFU/capsule	4 weeks	*Roseburia* and *Lachnospiraceae* ↑; *Verrucomicrobia* ↓	Attenuate exercise-induced intestinal hyperpermeability	[43]
*L. gasseri* CP2305	Male university students	5 × 10^7^ CFU/mL	12 weeks	*Faecalibacterium*, *Bifidobacterium* ↑	Alleviate fatigue/anxiety/depression, regulate blood hormones, modulate gut microbiota, and prevent stress-induced mitochondrial gene changes	[59],
*L. lactis* subsp. *lactis* LY-66, *L. plantarum* PL-02	Healthy men and women	7.5 × 10^9^ CFU/sachet	6 weeks	*Lactobacillus*, *Lachnospiraceae* ↑; *Sutterella* ↓ (PL-02) *Ruminococcus*, *Prevotella copri*, *Lactococcus lactis* ↑ (LY-66) *Akkermansia muciniphila* ↑ (PL-02 + LY-66)	Enhance exercise performance/muscle mass, increase beneficial bacteria, improve body composition/gut health	[56]

**Table 3:** The alterations in the composition of gut bacteria following the administration of plant polysaccharides and plant extracts.

Sources	Phytocompounds	Subject(s)	Dose	Duration	Changed Abundance of Gut Microbiota	Main Conclusion	Ref
Garlic slices	Garlic polysaccharide (GP)	Male ICR mice	1.25 and 2.5 g/kg-BW	7 weeks	*Alloprevotella*, *Muribaculum*, *Rikenellaceae_RC9*, *Parabacteroides*, *Dubosiella* and *Eubacterium_ventriosum_group* ↑; *Enterorhabdus* and *Desulfovibrio* ↓	Via AMPK/PGC-1α pathway: improve exercise fatigue, activate antioxidant capacity, modulate gut microbiota	[67]
*Polygonati rhizoma*	*Polygonati rhizoma* polysaccharides (PRP)	Male Kunming mice	250 mg/kg	7 days	*Akkermansia* and *Lactobacillus* ↑; *Streptococcus* ↓	Prolong mouse swimming time, improve blood glucose/antioxidant indices, modulate gut microbiota, alleviate EIF	[63]
Ginseng leaves	Fermented ginseng leaves	male Sprague-Dawley rats	50 mg/kg	4 weeks	*Bacteroidaceae*, *Allobaculum* and *Akkermansia* ↑	Elevate ginsenosides, improve fatigue-related biomarkers/gut microbiota, promote muscle cell repair	[27,64]
Ginseng	water extract of ginseng	male Sprague-Dawley rats	13 mg/kg	20 days	*X. Eubacterium. _* *ruminantium_group*, *Bifidobacterium* and *Clostridium_* *sensu_stricto_1* ↑	Promote SCFA production, improve glycogen storage, reduce inflammatory factors, and enhance anti-fatigue capacity	[33]
Konjac	Konjac glucomannan	Male C57BL/6 mice	1.25, 2.50, and 5.00 g/L as drinking water	42 days	*Prevotellaceae_Prevotella*, *Allobaculum* ↑; *Bifidobacterium* ↓	High-dose KGM: maintain microbial homeostasis; medium-dose: boost beneficial bacteria/SCFA production, enhance endurance/strength	[66]
*Tuber indicum*	*Tuber indicum* polysaccharide	Male C57BL/6 mice	0.1,0.3 and 0.9 g/kg body weight	40 days	*Porphyromonadaceae*, *Bacteroidaceae* and *Rikenellaceae* ↑; *Prevotellaceae*, *Ruminococcaceae* and *Helicobacteraceae* ↓	Reduce blood lactic acid, elevate ATPase activity, improve intestinal permeability, and modulate gut microbiota	[65]
/	Resveratrol	male ICR mice	50 mg/kg	30 days	*Megasphaera* and *Lactobacillus* ↑; *Brevundimonas diminuta* and *Coprobacillus* ↓	Prolong mouse endurance swimming time, improve serum indices, enhance intestinal barrier function, and modulate gut microbiota	[25]
Ginseng	water extract of ginseng	male Sprague-Dawley rats	1.42 g/kg	14 days	*Lactobacillus* and *Bacteroides* ↑; *Anaerotruncus* ↓	Regulate energy metabolism/antioxidation, ameliorate gut dysbiosis, alleviate EIF	[68]
*Brassica rapa* L.	aqueous extract of *Brassica rapa* L. (AEB)	male Swiss mice	0.5 and 1 g/kg body weight	30 days	*Firmicutes* ↑; *Bacteroides* and *Proteobacteria* ↓; *Enterococcus*, *Sphingomonas*, *Mucispirillum*, *Pseudomonas* ↓	Antioxidant effect; regulate energy metabolism/inflammatory response/gut microbiota; and alleviate EIF	[42]
Ppitaya	polyphenol extract of pitaya fruit	male C57BL/6 J mice	200 mg/kg/day	21 days	*Helicobacter pylori*, *Desulfovibrio* and *Eubacteria* ↓	Prolong endurance time, enhance antioxidant capacity, regulate basal metabolism, activate PI3K/Akt pathway, improve gut microbial diversity	[69]

## Data Availability

Data sharing is not applicable to this article.
